# Comparison between Intra-Articular Steroid Injection and Supra-Scapular Nerve Block in the Management of Frozen Shoulder

**DOI:** 10.12669/pjms.40.7.8531

**Published:** 2024-08

**Authors:** Zeeshan Khan Nazim, M. Farhan Farhat, Saleem Abbasi

**Affiliations:** 1 Zeeshan Khan Nazim MS-Orthopedic and Trauma Surgery, Ex-Postgraduate Resident, Department of Orthopedics, PIMS, Islamabad, Pakistan; 2 Farhan Farhat MS-Orthopedic and Trauma Surgery, Ex-Postgraduate Resident, Department of Orthopedics, PIMS, Islamabad, Pakistan; 3 Saleem Abbasi Epidemiologist, Pakistan Institute of Medical Sciences, Islamabad - Pakistan

**Keywords:** Frozen shoulder, SPADI, ROM, Supra-scapular nerve block, Intra-articular steroid injection

## Abstract

**Objective:**

To compare the efficacy of intra-articular steroid injection with ultrasound-guided supra-scapular nerve block in the management of frozen shoulder in terms of shoulder pain and disability index score and range of motion.

**Method:**

This randomized controlled trial was conducted in orthopedic department, PIMS, Islamabad from 1^st^ January, 2020 to 30^th^ June, 2020. A total of 72 patients were randomly equally (n=36 each) allocated to Group-A (intra-articular steroid injection) and Group-B (supra-scapular nerve block). Adults above 18 years of both genders having diffuse shoulder pain were included. Cases of shoulder pain localized because of bicipital tendinitis, rotator cuff tear, pain due to acute trauma and those with osteoarthritis were excluded. Data was analyzed in SPSS version 22.0.

**Results:**

Patients average age was 60.1 ± 6.29 in IASI and 58.0 ± 5.83 years in SSNB Group-And there were 19 (52.8%) males in IASI group compared to 15 (41.7%) in SSNB. At three weeks mean pain was significantly less in SSNB (57.1 ± 9.53 vs 49.4 ± 9.02) compared to IASI group (p-value, <0.001). The mean disability index was significantly low in SSNB (51.5 ± 5.10 vs 63.9 ± 5.14) versus IASI group (p-value, <0.001). At one week, three weeks and six weeks, mean external rotation was better in SSNB than IASI group (p-value, <0.001). The mean abduction was also better in SSNB group.

**Conclusion:**

Ultrasound guided supra-scapular nerve block is better than intra-articular steroid injection in managing frozen shoulder.

## INTRODUCTION

Frozen shoulder is a condition when shoulder becomes stuck and less mobile. It presents in the orthopaedic OPDs quite commonly. The prevalence of frozen shoulder also called adhesive capsulitis ranges from 6.7% to 66.7% in the general population.^1^ The muskoskeletal issue varies according to geographical areas and affected populations specially, those having less active life styles.^2^

The main cause of the severe shoulder complications and pain is frozen shoulder or adhesive capsulitis, which progresses to loss of function and finally transforms into low quality of life.^3^ The most common etiologies of that pain are conditions like degenerative disease affecting the glenohumeral and acromioclavicular joints. And also those affecting the supporting soft tissue structures in addition to inflammatory diseases such as rheumatoid arthritis (RA), seronegative spondyloarthropathies and crystal arthropathies.^4^

Initially for frozen shoulder physiotherapy is recommended which is quite effective in reducing low grade shoulder pain and enhancing range of motion. However, in established critical shoulder disorders, it seems of little benefit.^5^ Other proven effective treatment strategies include simple analgesia, NSAIDs, intra-articular steroid injection, hydraulic distension, MUA and surgery. All these therapeutic agents have their benefits and limitations according to specific conditions.

Intra-articular steroid injections and supra-scapular nerve block guided via ultrasound are found to be highly effective in patients with frozen shoulder.^5^,^6^ There are a few reports on comparison of these treatment strategies. A study by Abdel-Shafi ME and colleagues found out that after 12 weeks of treatment of intra-articular steroid injection and supra-scapular nerve block for frozen shoulder the Shoulder Pain and Disability Index (SPADI) pain score was 59.0 ± 11.0 and 49.0 ± 10.3 respectively.^7^

Another study by Verma DK et al reported that ultrasound-guided supra-scapular nerve block (24.0 ± 5.1) and intra-articular steroid injection (26.0 ± 4.3) were equal in the treatment of shoulder pain according to SPADI pain score.^8^ This shows that there is a variable outcome with these two interventions for the management of shoulder pain. Since frozen shoulders and pain not only affects a person’s daily routine and personal life but it has far reached effects for the national level as economic loss due to decreased quality of life.^9^

The treatment of frozen shoulder is multi-dimensional from physical exercise and life-style change to injections and surgical procedures.^10^ By way of evaluating the two therapeutic modalities i.e. ultrasound-guided supra-scapular nerve block and intra articular steroid injection, we had planned to find out whether they differ in terms of efficacy and safety.

There is very rare evidence on the management of shoulder pain from our local and national level settings. We had planned to conduct this randomized controlled trial to study the efficacy of ultrasound-guided supra-scapular nerve block and intra-articular steroid injection.

## METHODS

This randomized controlled trial was conducted in the Department of Orthopaedics Surgery, Pakistan Institute of Medical Sciences, SZABMU, Islamabad in a period of six months from 1^st^ January, 2020 to 30^th^ June, 2020. Adult patients above 18 years of age presenting with frozen shoulder were selected. Sample size was calculated using 95% confidence level, 5% level of significance, anticipated mean shoulder pain index in suprascapular nerve block group of 49.0 and in intraarticular steroid injection group of 59.0.^7^ The population standard deviation was 15.0. The sample size came out to be 36 cases in each group. A total of 72 shoulder pain patients were enrolled in this study divided equally by lottery method into group A (n=36) and Group B (n=36).

### Inclusion Criteria:

Adults above 18 years, of both genders having diffuse shoulder pain of more than four weeks, diagnosed clinically using symptomology and examination findings of limited range of motion, those agreeing to stop any analgesic medication one week prior to procedure and giving consent for participation were all included in the study randomly.

### Exclusion Criteria:

Patients with localized shoulder pain due to bicipital tendinitis, morbid obese, had rotator cuff tear, had pain due to acute trauma, those with juxtaglenohumeral joint fractures, post-surgery and those with bony deformity, patients with bleeding disorders and active infections, and patients who had any allergy or sensitivity to steroids or local anesthetics were all excluded.

### Ethical Approval:

It was taken for the study and a written informed was administered (No. F.1-1/2015/ERB dated 25-10-2019). Patients were screened from the outpatient orthopaedic department.

### Procedure:

***Intra-articular steroid injection:*** The steroid injection was infused by the researcher under supervision of an experienced orthopedic surgeon. Two milliliters of 40 mg/ml methylprednisolone with 2ml of 1% lignocaine was injected intra-articular in the affected shoulder through posterior approach, using traditional posterior arthroscopic portal landmarks using a 24G 1½ inch needle. After entering the joint, a negative aspiration was done and plunger pushed slowly with consistent pressure.

### Ultrasound guided suprascapular nerve block:

Ultrasound-guided nerve block was given by the researcher in collaboration with an experienced radiologist having experience in interventional radiology. Under aseptic precaution, real-time ultrasonography was performed with a 6-13 MHz linear-array ultrasound transducer. A 23G spinal needle was inserted in the longitudinal axis of the ultrasound beam to localize the tip of needle at the notch. Once needle was positioned, SSNB was given using four ml of 0.5% bupivacaine.

After the interventions, all study patients underwent physiotherapy sessions. They were assessed after one week while performing ROM exercise within the pain-free range of the shoulder. The primary outcome was Shoulder Pain and Disability Index (SPADI) and passive ROM of affected shoulder measured at one week, three weeks and six weeks.

### Statistical Analysis:

Data was entered and analyzed in SPSS software version 20.0. The continuous numerical variables like age, duration of symptoms, SPADI scores and ROM score were measured as mean and standard deviation. The categorical variables like sex, clinical presentation, and x-ray findings were analyzed as frequency and percentages.

Student’s t-test was used to compare the mean levels of pain score, disability score, total score, external rotation and abduction between the two study groups. A p-value of <0.05 was considered statistically significant.

## RESULTS

In this study, the mean age of patients was 60.1 ± 6.29 years in IASI group and 58.0 ± 5.83 years in SSNB group. There were 19 (52.8%) males in IASI compared to 15 (41.7%) in SSNB group. No difference was observed among study groups according to age and gender.

Most of the patients in SSNB group presented with right sided lateral pain while in IASI group majority came with left lateral pain. There were almost equal number of diabetic patients in both study groups; 15 (41.7%) in IASI and 14 (38.9%) in SSNB group. There were 11 (30.5%) patients in IASI group with muscle wasting whereas in SSNB group there were 7 (19.4%) cases with this condition. The mean duration of severity of symptoms in terms of acute pain was 6.7 days in ISAI group compared to 7.8 days in SSNB group. There was no statistical difference between the two groups in terms of presentation and duration of symptoms, (Table-I).

**Table-I:** Baseline and clinical characteristics of patients in two groups.

	IASI (n=36)	SSNB (n=36)	p-value
***Age (years)***			
Mean ± SD	60.1 ± 6.29	58.0 ± 7.83	0.15
***Gender***			
Male	19 (52.8%)	15 (41.7%)	0.34
Female	17 (47.2%)	21 (58.3%)	
***Laterality of pain***			
Right	10 (31.2%)	15 (41.7%)	0.37
Left	22 (68.8%)	21 (58.3%)	
***Diabetes mellitus***			
Yes	15 (41.7%)	14 (38.9%)	0.81
No	21 (58.3%)	22 (61.1%)	
***Shoulder appearance***			
Erythema	-	-	
Swelling	-	-	
Muscle wasting	11 (30.5%)	7 (19.4%)	0.32
***Duration of symptoms (months)***			
Mean ± SD	6.7 ± 3.0	7.8 ± 3.1	0.16

The average pain score according to SPADI tool was compared between the two group at baseline and different follow-ups. There was no difference in average pain levels between two groups at baseline (68.2 ± 8.15 vs 70.1 ± 5.88). At one week after intervention mean pain dropped more in SSNB group (60.1 ± 8.69 vs 56.6 ± 8.61), however, it was not statistically significant. At three weeks (57.1 ± 9.53 vs 49.4 ± 9.02) after intervention mean pain level was found significantly low in SSNB group compared to IASI group (<0.001). Similarly, at six weeks also the mean pain level was low in SSNB group.

The mean disability index according to SPADI score was slightly high in the SSNB group at baseline (p-value, <0.001). At one week post intervention the mean disability index was 63.9 ± 5.14 in IASI group compared to significantly low 51.5 ± 5.10 in SSNB Group and this difference in means was statistically highly significant (p-value, <0.001). Moreover, the mean disability (SPADI) was also significantly low in SSNB group at three week and six weeks after intervention (p-value, <0.001).

The total SPADI score was also found significantly low in the SSNB group after intervention. At one week after intervention the mean total SPADI score was 62.5 ± 3.68 vs 53.5 ± 5.11 and this difference in two means was statistically significant (p-value, <0.001). Similarly, the mean total SPADI score was significantly low in SSNB group at three weeks and six weeks (p-value, <0.001).

Furthermore, the range of motion (ROM) was compared between the two groups. The mean external rotation was similar between the two groups at baseline. At one week after intervention the mean external rotation was found significantly better in SSNB group than IASI group (50.9 ± 5.83 vs 43.3 ± 7.65). In the same way the external rotation was found significantly better in SSNB group at three weeks and six weeks after intervention (p-value, <0.001).

The mean abduction was almost similar between the two groups at baseline. At one week after intervention the mean abduction was found significantly better in SSNB group than IASI group (99.0 ± 4.90 vs 86.8 ± 14.3). In the same way the mean abduction was found significantly better in SSNB Group at three weeks and six weeks post intervention (p-value, <0.001), (Table-II).

**Table-II:** Comparison of patient outcome between two groups.

	IASI (n=36) (Mean ± SD)	SSNB (n=36) (Mean ± SD)	P-value
***Pain (SPADI)***			
Baseline	68.2 ± 8.15	70.1 ± 5.88	0.24
At 1 week	60.1 ± 8.69	56.6 ± 8.61	0.09
At 3 weeks	57.1 ± 9.53	49.4 ± 9.02	0.001
At 6 weeks	51.1 ± 8.80	44.6 + 8.85	<0.001
***SPADI***			
Baseline	67.9 ± 2.62	73.6 ± 2.36	<0.001
At 1 week	62.5 ± 3.68	53.5 ± 5.11	<0.001
At 3 weeks	59.5 ± 4.28	45.3 ± 6.01	<0.001
At 6 weeks	58.3 ± 4.21	40.3 ± 6.17	<0.001
***External rotation***			
Baseline	36.2 ± 5.26	35.8 ± 5.91	0.75
At 1 week	43.3 ± 7.65	50.9 ± 5.83	<0.001
At 3 weeks	47.7 ± 7.11	56.9 ± 5.11	<0.001
At 6 weeks	50.1 ± 10.1	60.0 ± 5.47	<0.001
***Abduction***			
Baseline	75.5 ± 11.0	70.9 ± 4.90	0.02
At 1 week	86.8 ± 14.3	99.0 ± 4.90	<0.001
At 3 weeks	89.0 ± 18.5	107.0 ± 5.12	<0.001
At 6 weeks	99.0 ± 12.9	114.1 ± 5.91	<0.001

## DISCUSSION

The present study highlights that the average pain score was significantly less in SSNB group than IASI group at one week, three weeks and six weeks post intervention for frozen shoulder. Similarly, the disability index (SPADI) was significantly less in the SSNB group compared to IASI. And the overall SPADI score was also found significantly better in SSNB patients.

There is ample evidence suggesting comparable evidence like the current study findings. A study by Shankar et al also proved ultrasound guided SSNB to be superior than anatomic landmark based injections in the relief of shoulder pain and movement.^11^ A pre and post intervention based local study by Iqbal MJ and colleagues reported high effect of supra-scapular nerve block in patients with frozen shoulder pain.^12^ Another local study also witnessed significant control in pain and marked improvement in symptoms of range of movement in supra-scapular nerve block group compared to intra-articular injection.^13^ The investigators inferred that the intervention with suprascapular nerve block produces faster and complete resolution of pain and also restores range of movement than those cases intervened with intra articular injection. The effect has been witnessed by many but the urgent and complete relief is hugely advantageous keeping in view the excruciating pain and compromised condition of frozen shoulder patients.

Another study by Mardani-Kivi M and colleagues also witnessed that Supra-scapular nerve block is an effective therapy with long-term pain relief and increased mobility of the shoulder joint in patients with adhesive capsulitis.^14^ SSNB has been found equivalent or superior for frozen shoulder when compared with other interventions too.^15^ Another trial by Abdelshafi ME and colleagues also witnessed that highly significant mean changes were achieved with suprascapular nerve block compared to intra articular steroid injection and also physical therapy (p=0.001) on the basis of SPADI pain, disability, total SPADI score and active movements.^7^ Wu WT and colleagues conducted a meta-analysis and revealed that single dose of corticosteroid-contained regimen given via ultrasound-guided posterior method is better practice of the capsular distension in patients presenting with frozen shoulder.^16^

There is also evidence which suggests that both SSNB and IASI are equal in the management of frozen shoulder. Verma DK et al reported that though there was a significant effect of SSNB and IASI within groups after intervention for frozen shoulder, however, they witnessed no significant difference between the two groups.^8^ However the current study validates previous findings regarding superiority of SSNB in the management of frozen shoulder than IASI.

The most frequent clinical feature on physical examination was muscle wasting in both study groups. A previous study by Abdel-Shafi ME et al also reported that muscle wasting was the most frequency clinical complaint in their patients with frozen shoulder.^7^ Many others have also witnessed that muscle wasting is relevant finding in patients with advanced stage of frozen shoulder.^17^,^18^

The average SPADI score and ROM index were assessed and compared between IASI group and SSNB group as per primary objective. It was noticed that most of the SPADI and ROM baseline parameters were equivalent at baseline. The within group improvement in SPADI score and ROM was highly statistically significant in both groups, proving both interventions highly effective. The abundance of scientific evidence regarding better coverage of ultrasound-guided SSNB in improving SPADI index and ROM in these patients mandates its usage in the orthopaedic clinics with confidence.

The present study has many advantages; firstly, it was a randomized controlled trial and in comparing two treatment modalities independent probabilities were involved and lesser chance of biasness was there. Secondly, a reasonable sample of 36 patients each with frozen shoulder were allocated to the two therapies. Thirdly, there were no side effects noted after the two interventions.

### Limitations:

The number of cases with frozen shoulder did not present as expected and the data collection period had to be revisited. Due to COVID-19 situation, few of the patients were difficult to follow and their condition was only confirmed via telephone call, there was no case of dropout though. It is suggested that after few replications of this study in other regions of the country, based on the scientific evidence SSNB should be brought in routine practice in orthopaedic clinics.

## CONCLUSION

Ultrasound guided supra-scapular nerve block is more effective than intra-articular steroid injection in the management of frozen shoulder.

### Author’s Contribution:

**ZKN** and **MFF** conceived, planned, collected data as well as wrote introduction and methods section.

**SA:** Conducted statistical analysis and prepared the manuscript.

All authors critically reviewed and approved the final manuscript.
